# Mapping the Potential Distribution of Ticks in the Western Kanto Region, Japan: Predictions Based on Land-Use, Climate, and Wildlife

**DOI:** 10.3390/insects12121095

**Published:** 2021-12-07

**Authors:** Kandai Doi, Takuya Kato, Iori Tabata, Shin-ichi Hayama

**Affiliations:** 1 Laboratory of Wildlife Medicine, School of Veterinary Medicine, Nippon Veterinary and Life Science University, Musashino, Tokyo 180-8602, Japan; tkato@nvlu.ac.jp (T.K.); hayama@nvlu.ac.jp (S.-i.H.); 2 Center for Environmental Studies, Co., Tachikawa, Tokyo 190-0022, Japan; ioritree@mac.com

**Keywords:** MaxEnt, wildlife distribution, tick distribution

## Abstract

**Simple Summary:**

Recently, the risk of tick-borne diseases (TBD) has drawn increasing attention from a public health perspective. Information about where ticks are distributed is important for the prevention of TBDs. In this study, we used the MaxEnt model to predict the potential distribution of six major tick species out of 16 tick species collected in the Kanto region, the central part of Japan, based on land-use, climate, and wildlife distribution, and to investigate the factors that contribute to each distribution of ticks. The distribution of raccoons contributed to the distribution of five tick species, and forest connectivity was a strong contributor to the distribution of all species.

**Abstract:**

Background: Tick distributions have changed rapidly with changes in human activity, land-use patterns, climate, and wildlife distributions over the last few decades. Methods: To estimate potential distributions of ticks, we conducted a tick survey at 134 locations in western Kanto, Japan. We estimated the potential distributions of six tick species (*Amblyomma testudinarium* Koch, 1844; *Haemaphysalis flava* Neumann, 1897; *Haemaphysalis kitaokai* Hoogstraal, 1969; *Haemaphysalis longicornis* Neumann, 1901; *Haemaphysalis megaspinosa* Saito, 1969; and *Ixodes ovatus* Neumann, 1899) using MaxEnt modeling based on climate patterns, land-use patterns, and the distributions of five common wildlife species: sika deer (*Cervus nippon* Temminck, 1838), wild boar (*Sus scrofa* Linnaeus, 1758), raccoon (*Procyon lotor* Linnaeus, 1758), Japanese raccoon dog (*Nyctereutes procyonoides* Gray, 1834), and masked palm civet (*Paguma larvata* C.E.H. Smith, 1827)). Results: We collected 24,546 individuals of four genera and 16 tick species. Our models indicated that forest connectivity contributed to the distributions of six tick species and that raccoon distribution contributed to five tick species. Other than that, sika deer distribution contributed to *H. kitaokai*, and wild boar distribution, bamboo forest, and warm winter climate contributed specifically to *A. testudinarium*. Conclusions: Based on these results, the dispersal of some tick species toward residential areas and expanded distributions can be explained by the distribution of raccoons and by forest connectivity.

## 1. Introduction

The distribution of ticks provides important information for vector control. Tick distributions are determined by a combination of climatic factors and host distributions [1]. However, changes in host distributions are frequently reported [2,3,4]. In particular, the expansion of wildlife populations to urban and peri-urban areas has been reported in many cities, and such wildlife is called “urban wildlife” (e.g., Berlin, Chicago, New York, Rome, and Tokyo) [5,6,7]. Bradley and Altizer [8] indicated that urban wildlife results from changes in land-use and human activity. Abandoned areas are becoming corridors for the spread of wildlife as well as parasites and pathogens.

Urban wildlife is an issue in Japan and is closely associated with changes in human activity. In particular, the distributions of medium- to large-sized wildlife are substantially influenced by humans, especially in the Kanto region, which is located in central Japan and includes the mega-city Tokyo. In the Kanto region, after the late 1800s, the populations of many wildlife species decreased due to overhunting as well as habitat destruction carried out for military purposes [4]. To address the drastic decreases in populations of large wildlife (sika deer (*Cervus nippon* Temminck, 1838), Japanese black bear (*Ursus thibetanus japonicus* Schlegel, 1857), and Japanese serow (*Capris capricornis* Temminck, 1844)), the government implemented protection policies. Urban areas destroyed by warfare have become sites of rapid urban development, with the population concentrated around Tokyo and the development of residential areas based on the New Town Plan. As a result, the landscape of the buffer zone between the mountains and the city known as *satoyama* was lost, and the suburbs of Tokyo became an intermediate landscape between the city and the mountains [9]. Furthermore, the extreme concentration of the population in the center of the capital city has led to depopulation and population aging in the suburbs and rural areas at the border between mountainous and urban areas. As the result of government-implemented wildlife-protection policies and the loss of the *satoyama* landscape, wildlife habitats have expanded into cities. In particular, medium-sized carnivores, such as the Japanese raccoon dog (*Nyctereutes procyonoides* Gray, 1834; hereafter, tanuki), are common urban wildlife in Japan. In recent decades, the distributions of two introduced species, the masked palm civet (*Paguma larvata* C.E.H. Smith, 1827) and the raccoon (*Procyon lotor* Linnaeus, 1758), expanded rapidly into cities [10]. In addition, wild boars (*Sus scrofa* Linnaeus, 1758) and Cervidae are appearing in urban areas in many countries [7,11,12].

**Figure 1:**
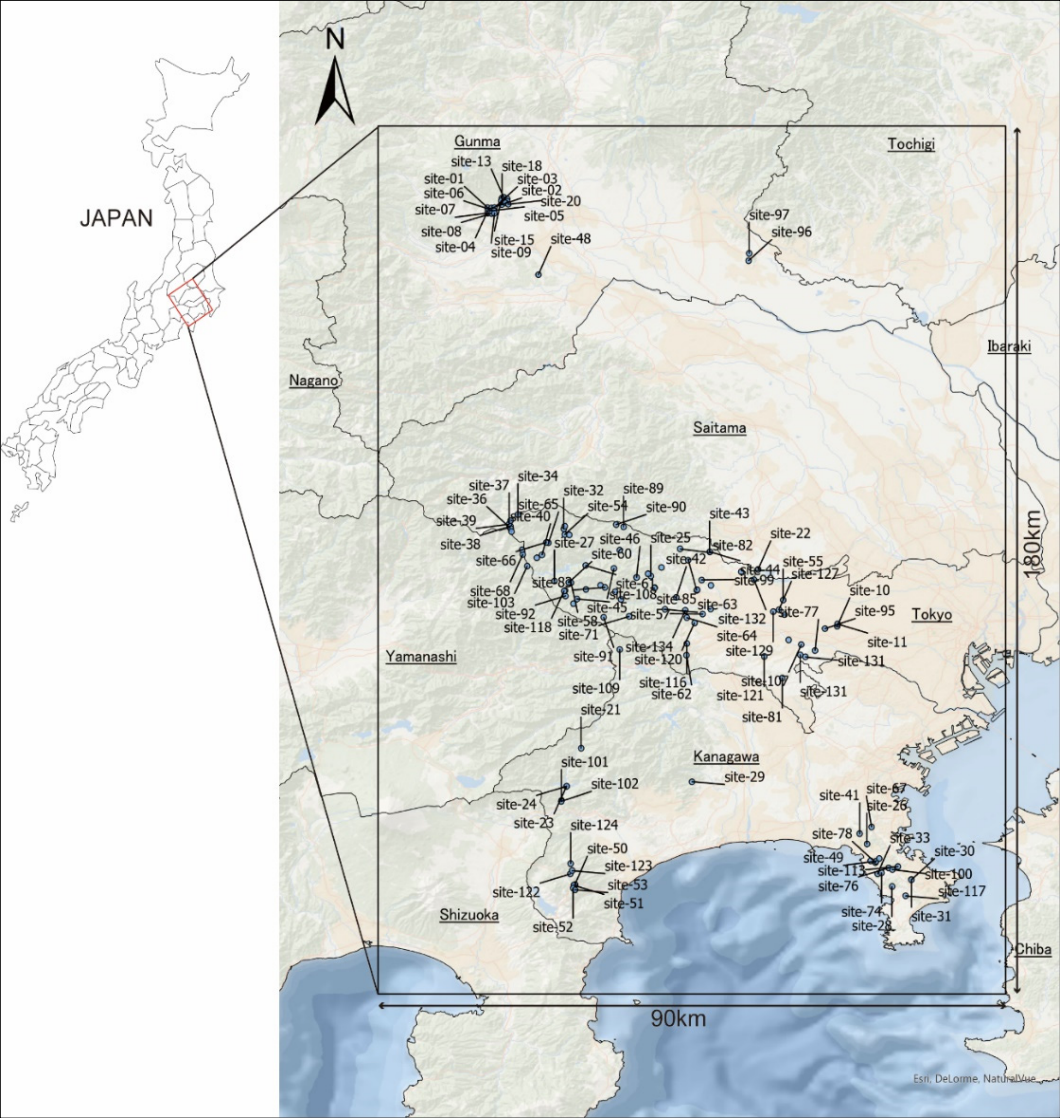
Study area (90 km × 180 km). The blue dots indicate tick survey sites (The basemap image is provided by Esri, Inc.).

Previous studies in the 1970s to 1990s have revealed the distribution of ticks to be at a large scale in Japan [13,14,15]. However, the changes in land-use patterns (i.e., abandoned agricultural areas), habitat fragmentation, and urban wildlife may have altered tick distributions dramatically in recent decades. In Japan’s Kanto region, tick-borne diseases (TBDs), such as Japanese spotted fever (JSF), are the most endemic types of diseases. JSF emerged in Tochigi Prefecture, Kanagawa Prefecture, Tokyo metropolis, Gunma Prefecture, and Nagano Prefecture in the last five years [16]. Lyme disease emerges sporadically in the Northwestern part of our study area (Figure 1) [17]. Severe fever with thrombocytopenia syndrome (SFTS) is not common in the Kanto region, but the SFTS virus antibody detected from preserved serum samples collected from a patient in 2017 was reported in Chiba Prefecture, which is a part of the eastern part of the Kanto region [18]. The preparation for the emergence and reemergence of TBDs requires updated information on tick distributions at high resolutions. Furthermore, this information needs to be linked to information on wildlife host distributions for the development of effective preventive measures against TBDs.

Here, we conducted a tick survey and estimated the potential distributions of major tick species in the western Kanto region of Japan using maximum entropy (MaxEnt) modeling [19], a machine-learning technique for species distribution models (SDMs). MaxEnt modeling estimates species distributions based on “presence” data for species and background factors, such as wildlife fauna, forest distribution, climatic patterns, and land-use patterns [20,21,22,23,24]. We estimated the potential distribution of major tick species based on our survey findings combined with land-use information and abiotic factors obtained by satellite images and government surveys. To improve our prediction, we applied the distribution patterns of the major wildlife in our study area as the distribution of available resources for ticks [25]. Our aim was to report the tick fauna in the western Kanto region and to determine the biological and abiotic factors contributing to vector tick distributions in urban to suburban areas and therefore to provide basic information for future TBD and vector control measures.

## 2. Materials and Methods

### 2.1. Study Area

Our study area was a 90 km × 180 km region spanning the Kanto Plain and the Kanto Mountains, including the Chichibu-Tama-Kai National Park, located in central Honshu Island (Figure 1). This region includes the coastline and peninsulas up to the alpine zone at an altitude of about 3700 m. The western part of the region is mountainous and covered mostly by forests, and the eastern part is occupied by agricultural land and urban areas from the plateau to the plains.

### 2.2. Tick Survey

We conducted a tick survey at 134 locations in the Tokyo metropolis, Kanagawa Prefecture, Gunma Prefecture, and Saitama Prefecture from January 2015 to December 2020 (Figure 1). Surveys in each prefecture were conducted for different purposes. We collected ticks using the flagging method with a 0.50 m × 0.75 m flannel fabric. We selected a day without rain to conduct our survey, and tick collection was conducted for at least 30 person-minutes for each survey and site. The collected ticks were preserved in a 70% ethanol solution until identification. The survey in the national park area was conducted under the authorization of the Kanto Regional Environment Office (authorization no. 2008216). The species, developmental stage, and sex of adults were determined based on morphological observations under a stereomicroscope and a compound microscope using identification keys [13,26,27]. We recorded the geographical coordinates of each tick survey point. After tick identification, we listed the “presence” locations of tick species for each survey point and projected the data in ArcGIS Pro [28].

### 2.3. Land-Use Factors

Ticks are not highly locomotive organisms, and their dispersion is often dependent on the movement of their hosts [29]. Thus, tick distributions may be affected by vegetation and anthropogenic activities such as agriculture and food waste, which attract wildlife hosts. Additionally, the land-use pattern may reflect microclimatic factors, which are formed by understory vegetation, and litter layer, which provide habitats for ticks [20,22,23]. We used a High-Resolution Land Use Land Cover Map Ver. 21.03, which is the satellite images with a resolution of 50 m obtained by the Japan Aerospace Exploration Agency (JAXA) [30]. The satellite images covered an area spanning 90 km east–west and 180 km north–south of the Kanto mountains and western Kanto Plain (Figure 1).

The land-use patterns were classified as water (WA), urban (UR), rice paddy (RP), cropland (CL), grassland (GL), deciduous broad-leaf forest (DBLF) (e.g., *Quercus* spp., *Acer* spp., *Populus* spp., *Fagus crenata* Blume, etc.), deciduous needle-leaf forest (DNLF) (e.g., *Larix kaempferi* Carrière), evergreen broad-leaf forest (EBLF) (e.g., *Camellia* spp., *Castanopsis* spp. *Cinnamomum camphora*, J. Presl, etc.), evergreen needle-leaf forest (ENLF) (e.g., *Cryptomeria japonica,* D. Don, *Chamaecyparis obtuse*, Endl., etc.), bare land (BL), and bamboo forest (BF). We also added solar panel (SP) areas because solar energy plant installation often involves logging, which may unintentionally lead to the creation of grassland and attract herbivores. We further assigned the land-use patterns to the broad categories forest area (FA), which included DBLF, DNLF, EBLF, ENLF, and BF, because such environments become covers for wildlife, and agricultural land (AL), which included RP and CL (Table 1). We calculated the area of each land-use category per 1 km^2^ geographical mesh. We also calculated the area difference between forests in a 2-km radius buffer circle and a 1-km^2^ geographical mesh to evaluate forest environment continuousness as forest connectivity (FC) [31]. We added digital elevation model (DEM) raster data provided by the Geospatial Information Authority of Japan and calculated the mean elevation of each 1-km^2^ geographical mesh as the elevation (EL) variable.

### 2.4. Climatic Factors

A climatic factor is known as the important determinant of a tick habitat. Randolph [24,32] reported temperature and moisture stress as important determinants of tick occurrence. For the climatic factors, we applied raster data for the lowest, highest, and mean temperatures of the coldest month (February) and the warmest month (August) as well as the mean snow depth as indicators of heat and cold stress in the tick population [33]. In addition, many species of ticks oviposit in the summer season [14]. The rains occur during June in Japan, and this may cause increasing of moisture stress during the reproductive period, which may affect the survival of eggs and larvae. Thus, we applied rainy season (June) precipitation (JP) as the humidity parameter in the reproductive season, and annual precipitation (YP) as the parameter considered for moisture stress in all developmental stages. All raster data were divided into a 1-km^2^ geographical mesh, and the mean value for each geographical mesh was calculated. The 1-km^2^ resolution raster data for five parameters (mean temperature of February, mean temperature of August, annual precipitation, June precipitation, and snow depth) were included as climatic factors in our models. All climatic data were provided by the National Spatial Planning and Regional Policy Bureau [34] (Table 1).

### 2.5. Wildlife Factors

We selected common mammalian wildlife lacking strict territories and non-solitary species in order to avoid overestimating the contribution to tick dispersion. The selected species were sika deer (WL1), wild boars (WL2), raccoons (WL3), tanuki (WL4), and masked palm civets (WL5) (Table 1). The distributional information was obtained from the Animal Distribution Atlas of Japan, which was provided by the Ministry of the Environment of Japan and modified based on recent data reported by the prefectures [35,36,37].

### 2.6. Tick Species Selection

The tick occurrence data were obtained from our tick survey. We assumed that a tick species is present in a geographical mesh when more than one specimen was collected, and then we generated a list of sites for each tick species. To avoid overestimation, species presence data with less than 20 recorded collection sites and less than two collected specimens from each survey point were excluded because ticks may be dropped off from wild birds and by human and companion animals traveling a relatively long distance and because ticks brought into an area have not distributed within the area yet. We also chose sites that we investigated more than three times in each season (Spring (March–May), Summer (June–September), Autumn (October–November), Winter (December–February)). The tick presence data were linked to GPS coordinates of each survey site and applied to the MaxEnt model for each tick species. The six species—*Amblyomma testudinarium* Koch, 1844; *Haemaphysalis flava* Neumann, 1897; *Haemaphysalis kitaokai* Hoogstraal, 1969; *Haemaphysalis longicornis* Neumann, 1901; *Haemaphysalis megaspinosa* Saito, 1969; and *Ixodes ovatus* Neumann, 1899—were included in the prediction model using MaxEnt 3.4.4. We used 75% of the distribution data as a training set and the other 25% as a test set [20,38].

### 2.7. Maximum Entropy (MaxEnt) Modeling

Before we applied all background data to MaxEnt 3.4.4 [19], we compared all factors to avoid overfitting in the models caused by multicollinearity [39]. We rejected the highest and lowest temperatures in February and August because of the high Pearson’s correlations (*r* > 0.8) with the mean temperature in February (FM) and August (AM). The 26 background factors were rescaled to cells with a resolution of 1 km^2^. Tick occurrences were regarded as occurring at the same resolution. The following setting was used for feature classes: “all feature classes”, 1 for the regularization multiplier. Additionally, 10,000 random points were created as pseudo-absence data [19,20,38]. We repeated the test 10 times with a 25% random test percentage. The wildlife distributions were installed as categorical data, and other background factors were installed as continuous data. The potential distribution of each tick species output by MaxEnt was projected in GIS. The suitability for ticks was estimated between 0.00 and 1.00, where values closer to 1.00 indicated more suitable conditions for ticks and values closer to 0.00 indicated unsuitable conditions for ticks. The area under curve (AUC) of the receiver operating characteristics curve (ROC) was determined to evaluate the model’s performance. AUC values of ≥0.7 were interpreted as adequate, ≥0.8 were interpreted as good, and ≥0.9 indicated an excellent performance [20]. We rejected the model when AUC < 0.7. Acceptable predictions were projected in GIS for comparisons of potential distributions among tick species by projecting the suitability values between 0.00 and 1.00; only values above the prediction threshold for “10 percentile training presence” calculated using MaxEnt 3.4.4 were projected on GIS [40]. We also conducted a jackknife test to evaluate the contribution and permutation importance of factors to the model.

## 3. Results

### 3.1. Tick Survey

**Figure 2:**
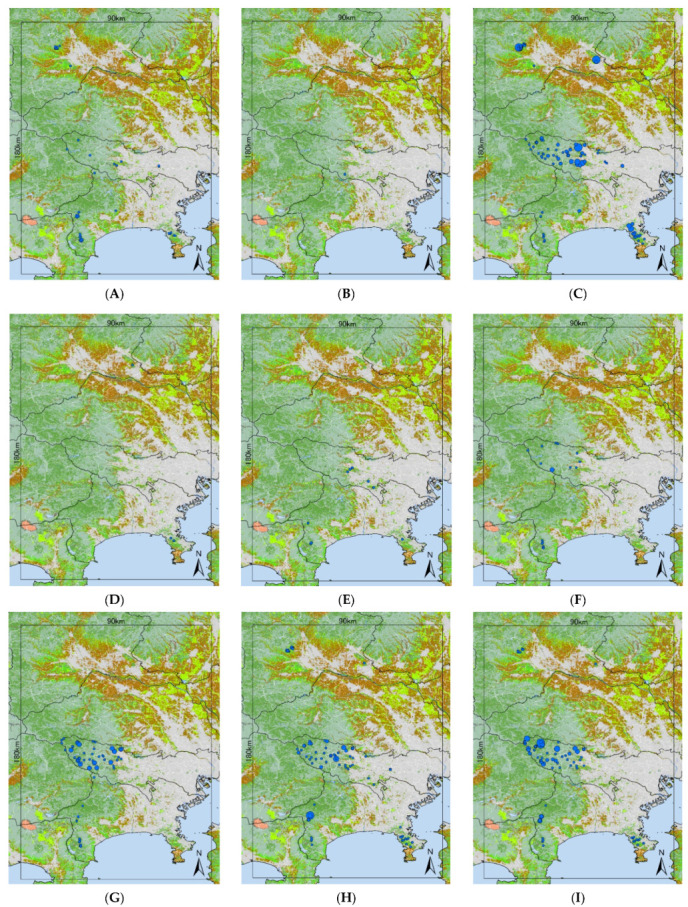

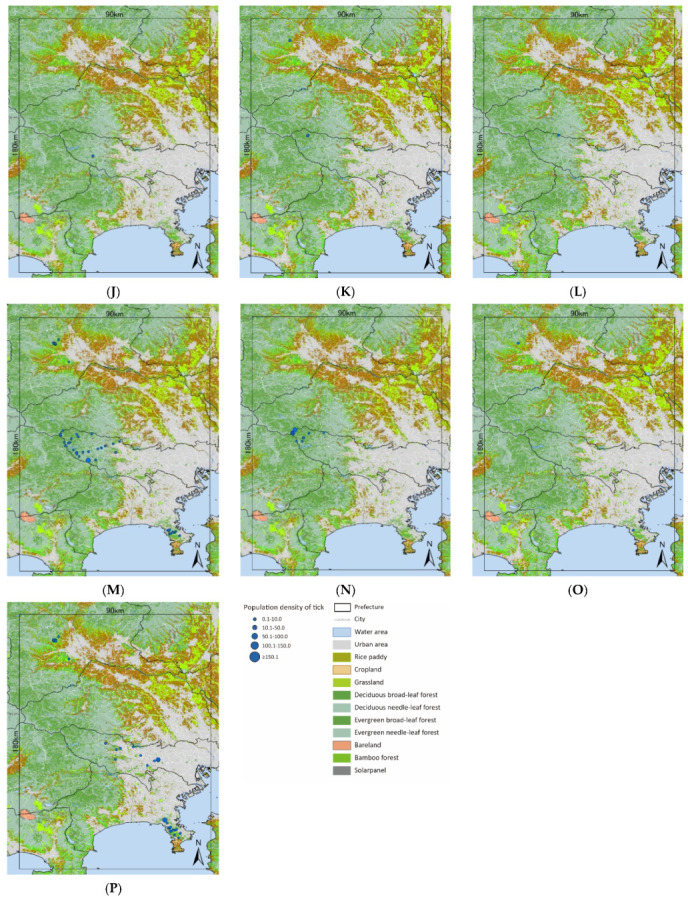
Tick collection sites for *A. testudinarium* (**A**), *D. taiwanensis* s.l. (**B**), *H. flava* (**C**), *H. formosensis* (**D**), *H. hystricis* (**E**), *H. japonica* (**F**), *H. kitaokai* (**G**), *H. longicornis* (**H**), *H. megaspinosa* (**I**), *I. columnae* (**J**), *I. monospinosus* (**K**), *I. nipponensis* (**L**), *I. ovatus* (**M**), *I. persulcatus* (**N**), *I. tanuki* (**O**), and *I. turdus* (**P**). The blue circle indicates where the species was collected, and the size of the circle indicates the population density of ticks.

We collected 24,546 individuals of 16 tick species: *A. testudinarium* (n = 345; 33 locations); *Dermacentor taiwanensis* sensu lato Sugimoto, 1935 (n = 2, 1 location); *H. flava* (n = 17,519; 118 locations); *Haemaphysalis formonensis* Neumann, 1913 (n = 3; 3 locations); *Haemaphysalis hystricis* Supino, 1897 (n = 25; 10 locations); *Haemaphysalis japonica* Warburton, 1908 (n = 73; 18 locations); *H. kitaokai* (n = 587; 61 locations); *H. longicornis* (n = 2406; 83 locations); *H. megaspinosa* (n = 2965; 84 locations); *Ixodes columnae* Takada and Fujita, 1992 (n = 1; 1 location); *Ixodes monospinosus* Saito, 1968 (n = 6; 3 locations); *Ixodes nipponensis* Kitaoka and Saito, 1967 (n = 15; 8 locations); *I. ovatus* (n = 118; 46 locations); *Ixodes persulcatus* Schulze, 1930 (n = 74; 13 locations); *Ixodes tanuki* Saito, 1964 (n = 5; 2 locations); and *Ixodes turdus* Nakatsuji, 1942 (n = 402; 36 locations). The locations of the tick collections are shown in Figure 2A–P. Detailed information for ticks collected at each survey site is shown in the Supplementary Materials (Table S1).

### 3.2. MaxEnt Models

We predicted the potential distributions of *A. testudinarium*, *H. flava, H. kitaokai, H. longicornis, H. megaspinosa,* and *I. ovatus* using MaxEnt modeling. We did not conduct modeling for *I. turdus* because the species is highly dependent on avian hosts and we lacked avian host distribution data [41]. The other four species were not included because they were collected at fewer than 20 sites.

**Figure 3:**
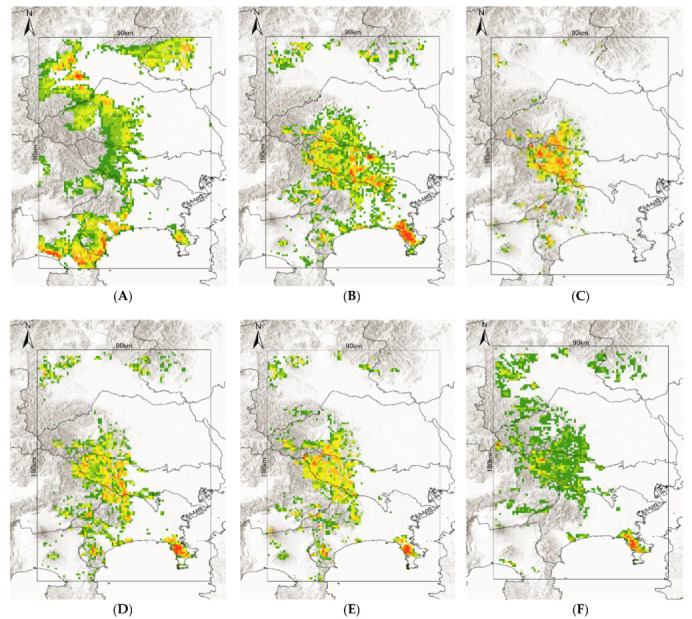
Potential distribution maps of *A. testudinarium* (**A**), *H. flava* (**B**), *H. kitaokai* (**C**), *H. longicornis* (**D**), *H. megaspinosa* (**E**), and *I. ovatus* (**F**). Red indicates a more suitable environment, and green indicates a less suitable environment for tick species.

**Figure 4:**
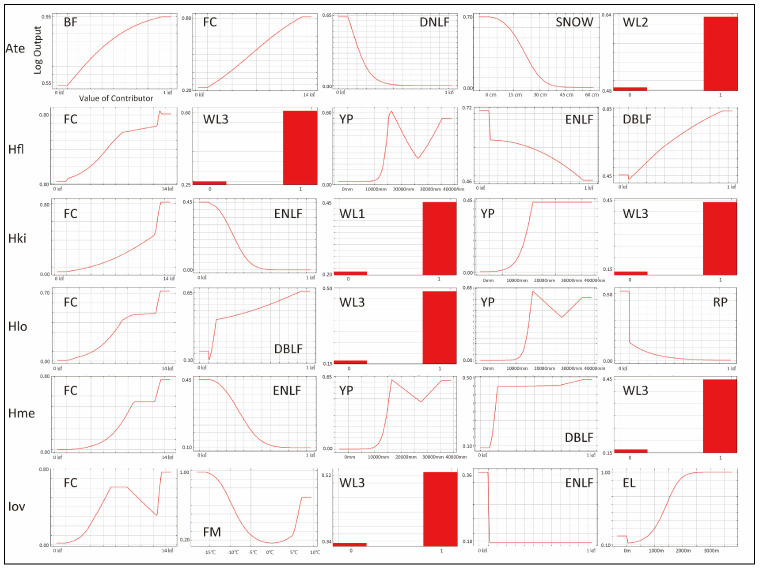
Response curves given by the logistic output for the values of the five variables with the greatest contributions for each tick species: *A. testudinarium* (Ate), *H. flava* (Hfl), *H. kitaokai* (Hki), *H. longicornis* (Hlo), *H. megaspinosa* (Hme), and *I. ovatus* (Iov). The *X*-axis indicates the value of the variable, and the *Y*-axis indicates the logistic output, which is the estimated probability of presence in the model. The relative impact for each variable is shown in Table 2.

All AUC values for the MaxEnt models were ≥0.7 (Table 2). The predicted potential distributions of the six tick species were projected on maps (Figure 3A–F). The response curves for the five variables with the greatest percentage contributions are shown in Figure 4.

- *A. testudinarium*
  The model performance for *A. testudinarium* was adequate (AUC = 7.00). The predicted distribution in Figure 3A indicated that suitable environments are spreading in the southwestern part of our study area, and a relatively lower suitability is shown at the base of the western mountainous area and in Tochigi Prefecture. The most important determinants of the *A. testudinarium* distribution were bamboo forest areas, forest connectivity, deciduous needle-leaf forest areas, snow depth, and wild boar distribution (Table 2). Collectively, the contribution of these variables to the model was 83.2%. The highest permutation importance was shown in the forest connectivity (PI = 46.3). Positive responses were observed for bamboo forest areas, forest connectivity, and wild boar distribution, while deciduous needle-leaf forest area and snow depth responded negatively for the habitat suitability of *A. testudinarium* (Figure 4).
- *H. flava*
  The model performance for *H. flava* was good (AUC = 0.84). *Haemaphysalis flava* has widespread suitable habitats in western Tokyo and Kanagawa Prefecture (Figure 3B). The most important variables for *H. flava* were forest connectivity, raccoon distribution, annual precipitation, evergreen needle-leaf forest areas, and deciduous broad-leaf areas. The tick distribution was positively influenced by forest continuity and raccoon distribution, and negatively influenced by evergreen needle-leaf forest areas and elevation. The response to annual precipitation was initially positive and then negative, which indicates that the habitat suitability increased with moderate precipitation (Figure 4). The cumulative contribution of these variables to the model was 73.2% (Table 2). The highest permutation importance was shown in forest connectivity (PI = 39.3).
- *H. kitaokai*
  The model performance for *H. kitaokai* was good (AUC = 0.88). *Haemaphysalis kitaokai* was predicted to be distributed in the mountainous areas of Tokyo metropolis, Kanagawa Prefecture, Saitama Prefecture, and Yamanashi Prefecture (Figure 3C). The most important variables for *H. kitaokai* were forest connectivity, evergreen needle-leaf forest areas, sika deer distribution, annual precipitation, and raccoon distribution. The highest permutation importance was shown in evergreen needle-leaf forest areas (PI = 21.8). The cumulative contribution of these variables was 86.7%. A negative response was only observed for evergreen needle-leaf forest areas, and the permutation impact for the model was the largest among the variables (PI = 21.8) (Table 2). Positive responses were observed for the other four variables. The cumulative contribution of these variables to the model was 73.4% (Figure 4).
- *H. longicornis*
  The model performance for *H. longicornis* was adequate (AUC = 0.79). The predicted distribution of *H. longicornis* was similar to that of *H. flava* but was skewed toward mountainous areas (Figure 3D). The most important variables for *H. longicornis* were forest connectivity, deciduous broad-leaf forest areas, raccoon distribution, annual precipitation, and rice paddy field areas. These variables showed a cumulative contribution of 79.9%. The highest permutation importance was shown in forest connectivity (PI = 49.3). Forest connectivity had the highest permutation importance to the model (PI = 49.3) (Table 2). Positive responses were observed for forest connectivity and raccoon distribution. Negative responses were observed for rice paddy field areas, and the response to annual precipitation was initially positive and then peaked between 1500 mm and 2000 mm (Figure 4).
- *H. megaspinosa*
  The model performance for *H. megaspinosa* was good (AUC = 0.83). The predicted distribution of *H. megaspinosa* was similar to the prediction for *H. longicornis*, but the suitable environment for *H. megaspinosa* included more mountainous areas (Figure 3E). The most important variables for *H. megaspinosa* were forest connectivity, evergreen needle-leaf forest areas, annual precipitation, deciduous broad-leaf forest areas, and raccoon distribution. The highest permutation importance for the model was forest connectivity (PI = 29.5) (Table 2). Forest connectivity, deciduous broad-leaf forest areas, and raccoon distribution showed positive responses. The response to annual precipitation was initially positive and then peaked between 1500 mm and 2000 mm. A negative response was observed for evergreen needle-leaf forest areas. The highest permutation importance was shown in forest connectivity (PI = 29.5), and the cumulative contribution of these variables was 82.1% (Table 2).
- *I. ovatus*
  The model performance for *I. ovatus* was excellent (AUC = 0.91). The relatively low suitability for *I. ovatus* was shown in the western Tokyo metropolis, western Saitama Prefecture, and southwestern Gunma Prefecture, while high suitability was shown in Miura Peninsula and Kanagawa Prefecture (Figure 3F). The most important variables for *I. ovatus* were forest connectivity, mean temperature in February, raccoon distribution, evergreen needle-leaf forest areas, and elevation. Forest connectivity had the highest permutation importance among the variables (PI = 45.7), and the cumulative contribution of these variables was 84.0% (Table 2). Positive responses were observed for raccoon distribution and elevation. A negative response was obtained for mean temperature in February up until 5 °C, and rapid positive responses were observed between 5 °C and 10 °C. Forest connectivity between 6 km^2^ and 15 km^2^ showed peaks in the log output (Figure 4). The contribution of these variables was 84.0% for the model of suitable environments for *I. ovatus* (Table 2).

## 4. Discussion

We collected individuals assigned to 4 genera and 16 species of ticks from 134 locations in 4 prefectures. To our knowledge, this is the first report of *H. hystricis* collected from the vegetation in Kanagawa Prefecture and Tokyo metropolis, of *H. formonensis* in Kanagawa Prefecture, and of *D. taiwanensis* s.l. in Tokyo metropolis. Although *H. hystricis* has been reported on hunting dogs in Tokyo [42], its distribution in the Kanto region and other northeastern parts of Japan needs to be updated. *H. formonensis* is distributed in the southwestern part of Honshu Island. *H. formonensis* was collected at limited sites on the peninsula in Kanagawa Prefecture, and its range may be wider than previously thought. *Haemaphysalis megaspinosa* is a relatively common species; however, it has not been reported in Gunma Prefecture. *Dermacentor taiwanensis* has been recorded in Kanagawa Prefecture [43]. Additionally, Apanaskevich and Apanaskevich [44] recorded *D. bellulus* in Kanagawa Prefecture. The classification of *D. taiwanensis* and *D. bellulus* Schulze, 1933, as separate species or as synonyms has been debated. We described our two specimens of *Dermacentor* tick as *D. taiwanensis* s.l., collected from vegetation in the Tokyo metropolis.

The potential distributions of ticks in many countries have been estimated by regression modeling and MaxEnt modeling [20,45]. The majority of these studies include climatic factors, soil pattern, land-use pattern, and forest types for determining the suitable habitat of ticks [20,46]. A previous study included soil patterns, such as pH [20]. In our model, we did not include pH distribution due to the lack of high-resolution data in our study area. The pH pattern would reflect the types of lower vegetation, which may reflect the habitat quality for ticks. Additional soil pattern data may increase the accuracy of the SDM for ticks. In addition, this study suggests that the forest connectivity highly contributed to the distribution of all six species. Forest connectivity in this study was calculated as the difference in the forest coverage within a circle with a radius of 2 km and a 1-km^2^ mesh. This is the indicator of how the forest patches, which serve as covers and habitats for wildlife, are connected. These connected forest patches provide a green corridor, which connects habitats and assists wildlife in expanding their distributions [4]. Therefore, the strong influence of forest connectivity in all models suggests that wildlife habitats increase tick host traffic and that questing ticks serve as host-searching areas.

Needle-leaf forest areas and deciduous broad-leaf forest areas were also contributors affecting multiple models. The evergreen needle forest in our study area consists mainly of *Cr. japonica* and *Ch. obtuse*. These trees do not provide fruits and have low vegetation due to the low solar radiation on the forest floor. Thus, evergreen needle-leaf forest areas are not likely to be selected by wildlife as foraging areas due to their low resource content. On the other hand, deciduous broad-leaf forest areas provide acorns and fruits, which are resources that attract wildlife. These characteristics of the forest type may reflect the negative response in evergreen needle-leaf forest areas and the positive response in deciduous broad-leaf forest areas for different species.

The following discussions are a description of the model variables for each tick species, especially the wildlife distribution variables in accordance with previous reports of tick collection from wildlife.

*Amblyomma testudinarium* is known as a species that is often collected from wild boars [47]. Ochi [48] reported that bamboo forests attract wild boars. Additionally, multiple reports have found immature *A. testudinarium* on a wide variety of wildlife, including small- to medium-sized mammals, reptiles, and birds [13,14,47,49]. Specifically, Yamauchi et al. [50] and Doi et al. [51] reported *A. testudinarium* infestation in medium-sized mammals such as tanuki, masked palm civets, and raccoons. This may suggest that an SDM for ticks that change its host preferences largely between developmental stages would perform better by splitting tick data based on their developmental stages. Additionally, Takada [14] explained that *A. testudinarium* is a species distributed in the southwestern part of Japan. This may explain our model for *A. testudinarium* showing negative responses in snow depth and deciduous needle-leaf forest areas. The deciduous needle-leaf tree in our study area is mainly *L. kaempferi,* which is distributed at higher elevations. These two variables suggest that *A. testudinarium* prefers environments with mild winters [14,46,49,51].

*Haemaphysalis flava* is a widely distributed species in Japan and other countries in Asia. Our model of the potential distribution of *H. flava* across the transitional area between the Kanto Mountains and the Kanto Plain suggests that a suitable environment is a region with a relatively high urban area coverage (Figure 3B). *Haemaphysalis flava* is the species infecting a wide range of medium and large mammals and wild birds but not small mammals [15,26,50,52]. Doi et al. [51,53] reported that *H. flava* was collected from raccoons, but masked palm civets could reduce tick infestation by grooming. Wild boars are also known as the main hosts for *H. flava* [47,54]. These reports explain the strong influence of raccoon distribution (15.2%) and wild boar distribution (4.8%) in the model (Table 2). The response curve of the annual precipitation suggested that moderate precipitation causes moderate humidity and provide a suitable environment for *H. flava*.

*Haemaphysalis kitaokai* is called the “deer tick” because its distribution often overlaps with the sika deer distribution [26,49,52]. Previous host records were mostly from Cervids [53]. In our model, sika deer distribution contributed 9.7%. This may be supported by the host specificity of *H. kitaokai*. The annual precipitation responded positively, which suggests that *H. kitaokai* prefer a humid area. Sika deer possibly also prefer moist environments. Yoshikawa et al. [55] reported that sika deer in mountainous regions are attracted to wetland vegetation. Thus, sika deer may frequently use areas with high precipitation to reach food resources, which caused annual precipitation to respond positively within our model (Table 2 and Figure 4).

*Haemaphysalis longicornis* is a generalist species. However, *H. longicornis* infestations on sika deer and correlations between questing height and sika deer height have been reported [56]. Tsukada et al. [57] suggested the abundance of adult *H. longicornis* is affected by the abundance of sika deer. On the other hand, immature stages of *H. longicornis* are known to infest medium- and large-sized mammals including raccoons, tanuki, masked palm civets, Japanese badgers (*Meles anakuma* Temminck, 1844), and wild boars [47,50,51,52,53,54]. The Miura Peninsula in Kanagawa Prefecture was projected to be a highly suitable environment for *H. longicornis* (Figure 3D). The sika deer is not distributed in this area, but wild boars, raccoons, tanuki, and masked palm civets are. These wildlife species may provide enough hosts for *H. longicornis* to become distributed, and the model did not suggest sika deer distribution to be a contributor with a high influence. Rice paddy field areas influenced the model slightly (9.9%), and the response curve indicated a negative response. Although rice cultivation is often damaged by sika deer and wild boars, ticks are reported to die in areas that are flooded [58]. *Haemaphysalis longicornis* is a species known to be active from early summer to autumn, and rice paddies draw water at this time of year, so RP likely reacted negatively due to the characteristics of ticks [49,59].

*Haemaphysalis megaspinosa* mainly infests sika deer but is also known to infest medium- and large-sized mammals such as Japanese black bears, wild boars, and raccoons [47,51,53,60]. However, Matsuyama et al. [61] reported a strong correlation between the abundances of *H. megaspinosa* and sika deer. The tick collection records in Figure 2I indicate relatively higher abundances of *H. megaspinosa* in the western Tokyo metropolis and western Kanagawa prefecture, while the abundances in Miura Peninsula and the southeastern Kanagawa Prefecture were not high. The MaxEnt model cannot consider the abundance of ticks. Using other models that can consider the abundance of the species, the habitat suitability may be better predicted.

*Ixodes ovatus* showed the largest distribution of not very suitable environments in our models. *Ixodes ovatus* is known to be distributed in the nest of rodents during their immature stages [14,27]. However, the flagging method can only collect *I. ovatus* at their adult stage. Adult *I. ovatus* are known to infest a variety of mammals (e.g., sika deer, wild boars, Japanese black bears, Japanese serows, raccoons, tanuki, masked palm civets, etc., [47,51,53]) and wild birds [41]. Our model suggested that raccoon distribution has the strongest influence (10.3%, Table 2). This tick was not abundant in our collection but was found to be widespread (Figure 2M). Fujimoto et al. [15] reported *I. ovatus* as a common *Ixodes* tick on many mammals and birds in the low-mountain region in Saitama Prefecture. The negative response for the mean temperature in February and the positive response for the elevation indicated that this species prefers cold weather [14]. *Ixodes ovatus* is also known to be distributed in Russia [14]. Thus, the species is highly resistant to cold environments. This may explain the low suitability in Figure 3F. The climate condition of the Kanto region is moderate in Honshu Island, Japan. This moderate climate may limit its habitat suitability, but the low host specificity may allow this species to be distributed in the Kanto region. The over-fitting of the MaxEnt model should also be considered, as it may be caused by the clustered distribution of collection sites. The accumulation of collected data in larger study areas including a greater variety of climate patterns and more uniformly distributed collection sites is needed.

As indicated above, raccoon distribution strongly influenced multiple species [51,54]. In particular, raccoon distribution contributed to the models for multiple tick species, with positive responses (Table 2 and Figure 4). Doi et al. [51,53] indicated that the raccoons in Kanagawa prefecture were infested by *H. flava* (all developmental stages), *H. longicornis* (all developmental stages), *H. megaspinosa* (all developmental stages), *H. japonica* (adult), *I. ovatus* (adult), *I. tanuki* (adult), and *A. testudinarium* (nymph and larva). The distribution of raccoons in the Kanto region is widespread from low mountain areas to urban areas. Additionally, raccoons are known to use similar ecological niches to tanuki and masked palm civets [62,63]. The tanuki distribution and the masked palm civet distribution did not strongly influence the models. The distributions of these two mammals almost cover all of our study area [35]. This may have caused an underestimation of their contribution to the model.

The sika deer distribution did not strongly contribute to *H. longicornis* and *H. megaspinosa*. This inconsistency between the models and the host specificity of the ticks may be avoided by creating a model for each developmental stage. We also suggest that the tick SDM model may need to consider the abundance and distribution of the host wildlife. Wildlife often show sparse, non-uniform distributions for various reasons, such as habitat size, herd formation, and resource distribution. Patterns of wildlife abundance may reflect the actual tick distribution and have better predictive values.

Based on the contributing factors and distributions, tick fauna are formed by wildlife host fauna, land-use patterns, and climate conditions, and the relative contributions of each factor differed among tick species. However, forest continuity and raccoon distribution were common denominators, with important contributions to multiple models [51,54]. Medium-sized mammals, including raccoons, Virginia opossums (*Didelphis virginiana* Kerr, 1792), and striped skunks, are associated with the emergence of tick-borne diseases and serve as dispersal vehicles for the spread of ticks to urban and suburban landscapes [64,65,66]. The raccoon is one of the most successful urban wildlife mammals [3]. Previous studies in Canada have shown that raccoons prefer habitats with both developed and natural areas, including forested and residential areas [65,66]. As a well-established host for the tick species, the raccoon distribution may affect the distribution of ticks via transport from the forest toward residential areas.

In addition, the estimated distribution patterns for *A. testudinarium*, *H. flava*, *H. kitaokai*, *H. longicornis*, *H. megaspinosa*, and *I. ovatus* were found along the foothills of the Kanto Mountains. Takada et al. [43] explained that the tick species collected in this study are vectors of TBDs. *Amblyomma testudinarium*, *H. flava*, *H. longicornis*, and *H. megaspinosa* are known to harbor the SFTS virus. *Haemaphysalis longicornis*, *H. hystricis, Haemaphysalis cornigera* Trapido and Hoogstraal, 1964, *D. taiwanensis* s.l., are the main vector species of *Rickettsia japonica*, the pathogen causing Japanese spotted fever. Lyme disease borrelia is harbored by *I. persulcatus* and *I. ovatus*. Our models showed that these vector ticks are relatively widespread near urban areas. Therefore, the results of this study encourage a more frequent monitoring for ticks and TBDs.

Estimations provided by other SDM methods, including MaxEnt modeling, are expected to be used for monitoring and controlling vector-borne diseases in the future. MaxEnt modeling often risks overestimation. However, in vector investigations with medical entomology, overestimation is preferred over underestimation. A certain number of false positives is acceptable, because it is important to not miss areas where vectors may be present if it leads to a risk of disease emergence. MaxEnt modeling is only able to project the habitat suitability or relative probability of species distribution but does not predict the actual probability of the presence of a species. However, SDMs such as MaxEnt are applicable to a wide variety of species, and as remote sensing data are available for public use, the MaxEnt model is probably one of the most feasible ways to obtain medical entomological data in a geographical perspective for future surveillance. Therefore, the predictions of this study cannot predict the direct risk of tick bites in humans and companion animals but are nonetheless valuable as advisory data to prioritize areas for further tick surveys.

## 5. Conclusions

We are the first to record that *H. hystricis*, *H. formonensis, H. megaspinosa*, and *D. taiwanensis* s.l. are distributed within our study area. Previous studies reported that these species are distributed in the southwestern part of Japan [13,14,17]. However, these species may have expanded their distributions into the Kanto region. We used MaxEnt modeling to estimate the potential distributions of six species of ticks. The factors with the greatest contributions to the models were forest connectivity and raccoon distribution. Invasive feral raccoons in the Kanto region have been an issue, and our results further suggest that raccoons are an example of urban wildlife carrying vector ticks of TBD pathogens. Our models suggest that continuous forest areas are becoming corridors for wildlife and that raccoon-like urban wildlife species are transporting ticks from these forests to residential areas. We conclude that the periodic surveillance of wildlife and ticks is necessary to obtain more precise information on the distribution of vector ticks. Additionally, machine-learning methods, such as MaxEnt modeling, are strong tools for obtaining distribution data from scattered information and for visualizing the risk of vector occurrence in map form, a universal interface, thereby providing essential information for novel solutions for vector control methods and wildlife management.

## Figures and Tables

**Table 1:** Land-use, climatic, and wildlife variables used as background factors for the prediction of tick distributions. All variables were obtained as raster data with a resolution of 1 km^2^ geographical mesh.

Code	Variable	Data Type	Unit	Range
UR	Urban area	Area-Continuous	km^2^	0.0–1.0
RP	Rice paddy area	Area-Continuous	km^2^	0.0–1.0
CL	Cropland area	Area-Continuous	km^2^	0.0–1.0
AL	Agricultural land area	Area-Continuous	km^2^	0.0–1.0
GL	Grassland area	Area-Continuous	km^2^	0.0–1.0
DBLF	Deciduous broadleaf forest area	Area-Continuous	km^2^	0.0–1.0
DNLF	Deciduous needleleaf forest area	Area-Continuous	km^2^	0.0–1.0
EBLF	Evergreen broadleaf forest area	Area-Continuous	km^2^	0.0–1.0
ENLF	Evergreen needleleaf forest area	Area-Continuous	km^2^	0.0–1.0
BF	Bamboo forest area	Area-Continuous	km^2^	0.0–1.0
FA	Forest area	Area-Continuous	km^2^	0.0–1.0
BL	Bare land area	Area-Continuous	km^2^	0.0–1.0
SP	Solar panel area	Area-Continuous	km^2^	0.0–1.0
WA	Water area	Area-Continuous	km^2^	0.0–1.0
FC	Forest connectivity	Area-Continuous	km^2^	−1.0–12.7
FM	Mean temperature in February	Temperature-Continuous	°C	−20.0–10.0
AM	Mean temperature in August	Temperature-Continuous	°C	10.0–30.0
YP	Annual precipitation	Precipitation-Continuous	mm	0–40,000
JP	Rain season (June) precipitation	Precipitation-Continuous	mm	0–40,000
SNOW	Snow depth	Depth-Continuous	mm	0–60
EL	Elevation	Height-Continuous	m	0–3776
WL1	Sika deer distribution	Present/Absent-Categorical	present (1)/absent (0)	0/1
WL2	Wild boar distribution	Present/Absent-Categorical	present (1)/absent (0)	0/1
WL3	Raccoon distribution	Present/Absent-Categorical	present (1)/absent (0)	0/1
WL4	Raccoon dog distribution	Present/Absent-Categorical	present (1)/absent (0)	0/1
WL5	Masked palm civet distribution	Present/Absent-Categorical	present (1)/absent (0)	0/1

**Table 2:** MaxEnt modeling results (AUC, SD, and Threshold), percentage contribution (%Cont), and permutation importance (PI) for each variable calculated in the jackknife test. The thresholds indicate the logistic output for which the species is estimated as being absent in the 1-km^2^ geographical mesh.

*A. testudinarium*			*H. flava*			*H. kitaokai*			*H. longicornis*			*H. megaspinosa*			*I. ovatus*		
AUC = 0.70			AUC = 0.84			AUC = 0.88			AUC = 0.79			AUC = 0.83			AUC = 0.91		
SD = 0.15			SD = 0.03			SD = 0.04			SD = 0.06			SD = 0.06			SD = 0.03		
Threshold = 0.37			Threshold = 0.26			Threshold = 0.37			Threshold = 0.32			Threshold = 0.30			Threshold = 0.36		
Variable	%Cont	PI	Variable	%Cont	PI	Variable	%Cont	PI	Variable	%Cont	PI	Variable	%Cont	PI	Variable	%Cont	PI
BF	37.6	2.1	FC	27.6	39.3	FC	48.4	17.9	FC	31.0	49.3	FC	40.2	29.5	FC	50.7	45.7
FC	12.7	46.3	WL3	15.2	7.7	ENLF	17.3	21.8	DBLF	14.8	10.3	ENLF	16.0	16.8	FM	11.2	2.9
DNLF	12.1	1.2	YP	14.1	14.0	WL1	9.7	3.9	WL3	12.8	9.8	YP	10.0	4.9	WL3	10.3	11.1
SNOW	10.5	25.3	ENLF	9.9	2.5	YP	7.2	4.5	YP	11.4	4.6	DBLF	8.2	9.2	ENLF	8.3	2.4
WL2	10.3	2.0	DBLF	7.8	5.6	WL3	4.1	2.1	RP	9.9	1.0	WL3	7.7	5.8	EL	3.5	9.6

## Data Availability

The data presented in this study are available in Table S1 (Please see Supplementary Materials).

## Supplementary Materials

The following are available online at https://www.mdpi.com/article/10.3390/insects12121095/s1, Table S1: The number of collected ticks per unit effort of flagging (60-min * person). Abbreviations: *Amblyomma testudinarium* (Ate), *D. taiwanensis* sensu lato (Dta), *H. flava* (Hfl), *H. formosensis* (Hfo), *H. hystricis* (Hhy), *H. japonica* (Hja), *H. kitaokai* (Hki), *H. longicornis* (Hlo), *H. megaspinosa* (Hme), *I. columnae* (Ico), *I. monospinosus* (Imo), *I. nipponensis* (Ini), *I. ovatus* (Iov), *I. persulcatus* (Ipe), *I. tanuki* (Ita), and *I. turdus* (Itu). The last letter indicates the developmental stage: adult (A), nymph (N), and larva (L) (for example, adult *I. ovatus* is written as “IovA”).
